# Shift from severe hypotension to salt-dependent hypertension in a child with autosomal recessive polycystic kidney disease after bilateral nephrectomies: a case report

**DOI:** 10.1186/s12882-023-03140-2

**Published:** 2023-04-04

**Authors:** Dovile Ruzgiene, Lauryna Abraityte, Karolis Azukaitis, Max Christoph Liebau, Augustina Jankauskiene

**Affiliations:** 1 Clinic of Pediatrics, Faculty of Medicine, Institute of Clinical Medicine, Vilnius University, Santariskiu Str. 4, Vilnius, Lithuania; 2 Department of Pediatrics, Center for Rare Diseases and Center for Molecular Medicine, University Hospital Cologne and Medical Faculty, University of Cologne, Kerpener Str. 62, Cologne, Germany

**Keywords:** Autosomal recessive polycystic kidney disease, Hypotension, Hypertension, Bilateral nephrectomy, Children

## Abstract

**Background:**

Autosomal recessive polycystic kidney disease (ARPKD) is a significant cause of morbidity and mortality in infants and children. In severe cases bilateral nephrectomies are considered but may be associated with significant neurological complications and life-threatening hypotension.

**Case presentation:**

We describe a case of a 17 months old boy with genetically confirmed ARPKD who underwent sequential bilateral nephrectomies at the age of 4 and 10 months. Following the second nephrectomy the boy was started on continuous cycling peritoneal dialysis with blood pressure on the lower range. At the age of 12 months after a few days of poor feeding at home the boy experienced a severe episode of hypotension and coma of Glasgow Come Scale of three. Brain magnetic-resonance imaging (MRI) showed signs of hemorrhage, cytotoxic cerebral edema and diffuse cerebral atrophy. During the subsequent 72 h he developed seizures requiring anti-epileptic drug therapy, gradually regained consciousness but remained significantly hypotensive after discontinuation of vasopressors. Thus, he received high doses of sodium chloride orally and intraperitoneally as well as midodrine hydrochloride. His ultrafiltration (UF) was targeted to keep him in mild-to-moderate fluid overload. After two months of stable condition the patient started to develop hypertension requiring four antihypertensive medications. After optimizing peritoneal dialysis to avoid fluid overload and discontinuation of sodium chloride the antihypertensives were discontinued, but hyponatremia with hypotensive episodes reoccurred. Sodium chloride was reintroduced resulting in recurrent salt-dependent hypertension.

**Conclusions:**

Our case report illustrates an unusual course of blood pressure changes following bilateral nephrectomies in an infant with ARPKD and the particular importance of tight regulation of sodium chloride supplementation. The case adds to the scarce literature about clinical sequences of bilateral nephrectomies in infants, and as well highlights the challenge of managing blood pressure in these patients. Further research on the mechanisms and management of blood pressure control is clearly needed.

## Background

Autosomal recessive polycystic kidney disease (ARPKD) is a significant cause of morbidity and mortality in infants and children. The phenotype of ARPKD can be very variable ranging from antenatal kidney function impairment to preservation of kidney function into adulthood [1]. Perinatal mortality from ARPKD is estimated to be approximately 30% with the most common cause being respiratory failure due to pulmonary hypoplasia [2]. In life-threatening cases, bilateral nephrectomies are considered but very early bilateral nephrectomies have been associated with significant hazards such as neurological complications and hypotension. The mechanisms of post-nephrectomy hypotension are not yet fully elucidated with fatal outcomes reported in severe cases [3–5].

We present a case report of an ARPKD patient after bilateral nephrectomies who developed severe post-nephrectomy hypotension with subsequent reversal to salt-dependent hypertension.

## Case presentation

The patient was transferred to our clinic from another center at the age of 10 months. Available history revealed that antenatal work-up showed hyperechogenic kidneys, pulmonary hypoplasia and progressive anhydramnios, thus labor was induced at 33 weeks of gestation (birth weight 2534 g). Genetic testing for suspected ARPKD was carried out and showed combined heterozygous variant in the *PKHD1* gene (p.Met2804Lys/p.Arg3482Cys). Both these variants in the InterVar database are evaluated as likely pathogenic/pathogenic, while in HGMD database are described as causing ARPKD. In addition, in silico analysis with the SIFT (Sorting Intolerant From Tolerant) tool indicated the mutation to be pathogenic (SIFT score 0.004). Due to extremely large kidneys, significant respiratory distress and feeding problems, the patient underwent sequential bilateral nephrectomies without adrenalectomies (at 4 and 10 months of age; total combined kidney mass—1.7 kg) and was started on continuous ambulatory peritoneal dialysis (PD). At admission the patient had severe hyperkalemia (exceeding 7 mmol/l) despite continuous resin therapy, hyponatremia and blood pressure (BP) on the lower range. Although laboratory evidence was lacking (low renin < 0,5 mU/L, elevated aldosterone 618 ng/l, but normal ACTH 27 ng/l levels) the patient was suspected to have functional adrenal insufficiency and fludrocortisone was initiated empirically. He was also switched to continuous cycling PD (CCPD) with 2.27 glucose solution, fill volume of 250 ml (35 ml/kg), the last fill of 150 ml, 12 cycles in total, UF 500 ml, and continued therapy with oral sodium chloride (7,5 mmol/kg) and calcium polystyrene sulphonate. Sodium chloride was not added to PD fluid. Following the initiation of fludrocortisone, his potassium levels normalized and BP remained on the lower range without significant hypotension. Liver work-up showed elastographic changes consistent with F2-F4 fibrosis with normal liver function and no signs of portal hypertension. After successful PD training the patient was discharged home with stable condition.

**Fig. 1:**
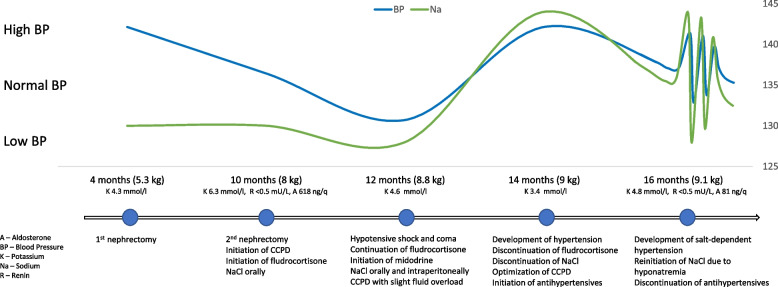
Schematic timeline of patient’s BP, clinical course and management

At the age of 12 months after a few days of poor feeding at home the boy experienced a severe episode of hypotension with shock and coma. He was resuscitated and transferred to our hospital on mechanical ventilation and high doses of vasopressors, and Glasgow Coma Score of three. Neurosonoscopy showed intact cerebral perfusion. Brain MRI showed a small hemorrhage in the right claustrum, cytotoxic cerebral edema and diffuse cerebral atrophy. During the subsequent 72 h the patient developed seizures requiring anti-epileptic drug therapy, gradually regained consciousness and was extubated. After discontinuation of vasopressor therapy, the boy remained significantly hypotensive and thus received high doses of sodium chloride (5,5 mmol/kg per day orally and 7,5 mmol/liter dialysate) with midodrine hydrochloride (1.25 mg) according to his BP. His BP further remained highly dependent on the amount of ingested fluids and sodium chloride receival, thus his UF was targeted to keep him in mild-to-moderate fluid overload. After a few months the boy started to develop hypertension (BP levels exceeding 160/100 mmHg). Fludrocortisone was discontinued with no significant impact on BP. Repeat adrenal function tests were normal (aldosterone 81 ng/l, ACTH 10 ng/l), renin levels remained unmeasurable and thyroid function was normal. Echocardiography and fundoscopy revealed no evidence of hypertension-mediated organ damage. The patient initiated antihypertensive therapy requiring four medications. After optimizing PD to avoid fluid overload and discontinuing sodium chloride, the patient was finally taken off antihypertensives but severe hyponatremia leading to hypotensive episodes occurred. Sodium chloride (3,5 mmol/kg) was reintroduced to maintain sodium at least on the lower range resulting in recurrent salt-dependent hypertension (BP up to 130/90 mmHg). The trajectories of BP, clinical course and management of the patient are schematically summarized in Fig. 1.

The patient is now 17 months old on home CCPD, on chronical kidney disease (CKD) medications including calcium-based phosphate binders, alphacalcidol, cholecalciferol, erythropoietin and folic acid. His weight is 9.3 kg (< 3^rd^ centile) and height is 75 cm (< 3^rd^ centile). His psychomotor development is delayed by 8–9 months. His BP remains in the 100/55 – 130/85 mmHg range while on sodium chloride, being lower after the dialysis.

## Discussion

We present a case of an atypical post-nephrectomy BP course in an ARPKD patient who underwent bilateral nephrectomies and developed life-threatening post-nephrectomy hypotension with subsequent reversal to salt-dependent hypertension.

The characteristic features of ARPKD are nephromegaly and pulmonary hypoplasia. These anomalies may impair respiratory function, while nephromegaly may restrict the diaphragm's movements and presses on the gastrointestinal tract, thus interfering with infant feeding. Nephrectomy is sometimes used as a therapeutic approach under the assumption to facilitate gastrointestinal function and pulmonary expansion, secure space in the abdominal cavity for PD and/or control hypertension [5]. While unilateral nephrectomy is attempted in some patients, need for subsequent contralateral nephrectomy has been reported in some cases due to increasing residual kidney volume, persistent respiratory distress or persistent hypertension. Some authors suggested that early bilateral nephrectomies in ARPKD may be associated with shorter hospital stays and parenteral nutrition duration [6]. However, bilateral nephrectomies are associated with the need of early kidney replacement therapy (KRT) which must be carefully weighed against presumed benefits and the evidence for benefits of the interventions is limited to few studies mainly focusing on nutritional aspects [7].

Both, bilateral nephrectomies and PD itself are associated with increased risk of severe hypotension [3]. Hypotension has been described in children undergoing bilateral nephrectomies for Denys-Drash syndrome [3], congenital nephrotic syndrome [4, 8], ARPKD [9, 10], neoplastic diseases (epithelioma, bladder carcinoma) and hydronephrosis [9]. The data regarding the course of and risk factors of post-nephrectomy hypotension is limited. In a retrospective observational study of 51 subjects who underwent 42 unilateral, 4 two-stage bilateral and 5 simultaneous bilateral nephrectomies by Nishi et al., eight patients experienced 11 post-nephrectomy hypotension events. In the same study, pre-nephrectomy hypertension was a significant risk factor of subsequent hypotension [5]. Liu et al. described hypotension that occurred shortly after bilateral nephrectomies in two patients with no previous high BP. In one of them hypotension persisted for six years until the BP rose again, while in the other, the BP did not recover during the limited follow-up time available. Hypotension treatment generally consists of fluid optimization – maintaining light fluid overload and the administration of sodium chloride or midodrine hydrochloride. We have observed that oral and intraperitoneal sodium chloride administration with midodrine during hypotensive episodes helped to maintain BP in the normal range in our patient, but fluid-based approach was not successful. Yet, response to fluid-based therapeutic interventions after bilateral nephrectomies can be very variable and some patients do not demonstrate response.

The mechanisms of post-nephrectomy short-term hypotension are not fully elucidated but mainly relate to impairment of the circulating renin-angiotensin system (RAS) [9]. However, further research on long-term post-nephrectomy changes in circulating RAS is clearly needed. According to Ferrario et al. findings in anephric rats, plasma angiotensin-II concentration dropped by 66% 48 h after bilateral nephrectomies, indicating that removal of the renal source of circulating renin is associated with a prominent fall in plasma angiotensin-II concentration [11]. Liu et al. reported transient hypotension with very low plasma renin concentrations, but normal plasma angiotensin-II concentrations after bilateral nephrectomy. We have observed similarly decreased renin levels with elevated aldosterone. These results suggest kidney and plasma renin independent production of angiotensin-II. For instance, the vascular system, adipose tissue, the brain and adrenal glands have been suggested as potential alternative sites of extrarenal RAS components production [9].

Very early bilateral nephrectomies are also associated with severe neurological complications, such as seizures, ischemic lesions, hypoxic brain injury, cerebral infarction, parenchymal defect, neurodevelopmental impairment and optic neuropathy with loss of vision [3, 4, 8, 10]. Data from ARegPKD study showed that patients after very early bilateral nephrectomies (within the first three months of life) had a fivefold increased risk of severe neurological complications. Very early bilateral nephrectomies are thought to be highly disruptive to blood pressure autoregulation, as plasma renin and aldosterone levels have been shown to peak in the first three months of life and then decline thereafter [12]. Furthermore, episodes of hypotension have been documented as independent risk factors for the observed severe neurological complications [10, 12]. In line with these findings, our patient experienced global cerebral ischemia following a severe hypotension episode. Importantly, early restoration of BP with vasoactive medications allowed full recovery regaining complete former psychomotor development, but in general his neurodevelopment is retarded by 8–9 months.

Arterial hypertension is a common complication in children with ARPKD [13, 14] and other chronic kidney diseases [15]. Unilateral or bilateral nephrectomies have been suggested as a potential therapeutic approach in patients with severe or refractory hypertension with relatively favorable outcomes reported by some authors [16] but evidence remains limited. On the contrary, there are reports describing recurrent or persistent hypertension after bilateral nephrectomies. Ajlan et al., report of a 6 years old patient with ARPKD who developed recurrent arterial hypertension after bilateral nephrectomies, which was performed as a last treatment option for refractory hypertension [15]. Liu et al. also described a patient with high arterial blood pressure prior to bilateral nephrectomies, which recurred 4 years postoperatively [9]. On the other hand, volume-mediated hypertension has been reported in patients after nephrectomy that can be managed successfully with intensified dialysis [17]. Overall, it is very difficult to balance between fluid overload and restriction after bilateral nephrectomies, as it was in our case. Moreover, hypertension in our patient was salt-dependent but sodium chloride dose could not be lowered due to recurrent hyponatremia.

## Conclusions

Our case describes severe hypotension and subsequent development of salt-dependent hypertension following bilateral nephrectomies highlighting the complex disruption of BP control mechanisms in such clinical circumstances. Although no clear mechanistic explanation (e.g., related to the RAS system or fluid control) can be derived from our case, it adds to the scarce literature about clinical sequences and management difficulties following bilateral nephrectomies in infants with ARPKD. Further experimental and observational studies are required to describe and explain the mechanisms and phenotypes of BP control after bilateral nephrectomies in this unique population.

## Data Availability

Not applicable.

## Abbreviations

ACTH: Adrenocorticotropic hormone
ARPKD: Autosomal recessive polycystic kidney disease
BP: Blood pressure
CCPD: Continuous cycling peritoneal dialysis
CKD: Chronic kidney disease
KRT: Kidney replacement therapy
MRI: Magnetic resonance imaging
PD: Peritoneal dialysis
RAS: Renin-angiotensin system
UF: Ultrafiltration
